# Engineering Alginate Membranes With Green Propolis‐Coated Silver Nanoparticles for Potential Use in Wound Dressing

**DOI:** 10.1002/cbdv.202503404

**Published:** 2026-07-06

**Authors:** Erica Tirzah S. Lima, Victoria L. S. Santos, Jad Lorena F. Simplício, Erika S. Lisboa, Sona Jain, Eliana B. Souto, Patrícia Severino

**Affiliations:** ^1^ Institute of Research and Technology University Tiradentes Aracaju Sergipe Brazil; ^2^ UCD School of Chemical and Bioprocess Engineering University College Dublin Dublin Ireland; ^3^ Center For Sciences of Imperatriz Federal University of Maranhão – UFMA Imperatriz Maranhão Brazil; ^4^ Department of Morphology Federal University of Sergipe São Cristóvão Sergipe Brazil

**Keywords:** alginate membranes, green propolis, silver nanoparticles, water vapor permeability, wound dressing

## Abstract

This study proposes a novel polymeric membrane composed of sodium alginate incorporating silver nanoparticles (AgNPs) and green propolis extract (GPE), for potential wound dressing. Five formulations were developed: a control (MB, alginate/glycerol), one with GPE at 0.5 mg/mL (MBE), and three with increasing silver nitrate (AgNO_3_) concentrations of 1.0, 3.0, and 5.0 mM (MBE1, MBE3, and MBE5). AgNP formation was confirmed by a visible color shift from white to dark brown. Characterization via FT‐IR, DSC, and TGA/DTG confirmed successful incorporation of GPE and AgNPs into the polymeric matrix, revealing molecular interactions and distinct thermal stability profiles across all membranes. Membrane thickness increased progressively (0.118–0.174 mm) with AgNP concentration. Water vapor permeability (WVP) improved upon adding GPE and AgNPs, peaking at 1.47 × 10^−^^3^ g.mm/kPa·s·m^2^ for MBE3. Mechanically, AgNP‐containing membranes exhibited greater flexibility, with strain values near 90% and Young's Modulus between 0.29–0.32 MPa. Wettability analysis showed increased hydrophilic character of AgNP membranes, with contact angles as low as 34.3°. Biocompatibility testing using the chorioallantoic membrane (CAM) assay confirmed non‐irritating profile. Molecular docking of GPE's major compounds—drupanin, artepilin C, and 4,5‐dicaffeoylquinic acid—further supports the therapeutic potential of these dressings for wound care applications.

## 1 Introduction

Chronic wounds can result from a variety of causes, including burns, traumatic injuries, pressure ulcers, diabetic ulcers, and surgical procedures, all of which contribute to a gradual and often debilitating loss of tissue [1]. Each year, millions of people around the world face the complex challenges posed by chronic skin lesions, with approximately 37 million people affected worldwide. These troubling figures reflect not only a significant decline in the health and quality of life of those affected, but also a significant economic burden due to the prolonged duration of treatment and frequent need for hospitalization [2]. The presence of microorganisms, such as *Staphylococcus aureus*, *Escherichia coli*, and *Candida albicans*, is common in such wounds, where secondary infections can further complicate the healing process. These infections often result in increased local pain, accumulation of serous or purulent exudate, crusting, and a marked reduction in the effectiveness of therapeutic interventions [3].

Recent advances in the treatment of skin lesions have emphasized the technological development of biomaterials, which play a critical role in enhancing the healing process. These biomaterials are used for the production of various devices, including membranes, subdermal implants, dressings, and nanoparticles, all of which provide structural support and allow for the controlled release of bioactive substances, thereby improving therapeutic outcomes [4].

Among these biomaterials, natural polymeric membranes, particularly those made of collagen, chitosan, and alginate, have gained attention due to their accessibility, biocompatibility, low toxicity, and efficacy in wound healing [5, 6]. Alginate, a polysaccharide derived from seaweed, is particularly valued for its ability to form a protective gel that efficiently absorbs exudate, and promotes tissue regeneration, making it ideal for the treatment of deep ulcers, burns, and pressure ulcers. In addition, these membranes can be enhanced with antimicrobial agents, such as silver nanoparticles (AgNPs). The inherent porosity and hydrophilic nature of alginate allow for the controlled release of these antimicrobial agents, significantly increasing their therapeutic efficacy [7].

The properties of alginate can be further enhanced by incorporating bioactive compounds, such as green propolis extract (GPE), which has been widely used for wound treatment [8]. Due to the complex and variable chemical composition of green propolis, antimicrobial, and anti‐inflammatory activities have been reported in the literature [9]. The therapeutic potential of green propolis is attributed to its flavonoids, phenolic acids, aldehydes, and ketones [10].

To further optimize the properties of membranes for use as wound dressings, the incorporation of additional antimicrobial compounds has emerged as an effective strategy. Among these, green synthesized (AgNPs) are considered a promising option [5]. AgNPs can be obtained in situ (i.e. within the delivery system), and exhibit a wide range of properties, including antiviral, antifungal and antiparasitic, and antimicrobial activity against both Gram‐positive and Gram‐negative bacteria. The biological synthesis (or biosynthesis) of AgNPs using GPE is an innovative, safe, sustainable, and cost‐effective technique to generate these particles within the delivery system. In biological synthesis, silver ions from silver nitrate (AgNO_3_) are reduced and stabilized by the bioactive compounds present in green propolis [11, 12]. Moreover, it is important to note that AgNPs show low toxicity to human cells, making them even more attractive for biomedical applications [13, 14, 15].

This study gains importance due to the limited research in the literature addressing the use of alginate membranes incorporated with GPE‐coated AgNPs for wound healing. The innovation of this work stems from the synergistic combination of the antimicrobial, wound healing and regenerative properties of sodium alginate and AgNPs with the bioactive compounds of GPE, offering a promising and effective therapeutic strategy. Given the high toxicity and adverse effects of conventional treatments, besides the risk of microbial resistance coupled with the substantial financial costs, the developed GPE‐coated AgNPs‐composed sodium alginate membranes are a low‐cost, biocompatible alternative with controlled release properties.

## 2 Materials and Methods

### 2.1 Materials

Sodium alginate (ProtonalRF6650) was purchased from FMC Corporation (Philadelphia, PA, USA), glycerol (≥99.5% purity), AgNO_3_, and all other reagents were bought from Sigma–Aldrich (St. Louis, MO, USA). Green propolis was kindly donated by the Federal University of Santa Catarina (Florianópolis, Santa Catarina, Brazil). Filtered double distilled water was home supplied using a Millipore system (Millipore GmbH, Burlington, MA, USA).

### 2.2 Production of AgNPs‐Composing Alginate/Glycerol Membranes

To obtain AgNPs‐composed alginate/glycerol membranes, firstly sodium alginate and glycerol were solubilized in water at the concentration of 4% and 1% (m/v), respectively, followed by homogenization for 24 h at 500 rpm with a mechanical stirrer Kasvi K40‐182OH (Pinhais, Paraná, Brazil), to prepare a polymeric solution. The GPE, obtained as previously described by us [16], was then incorporated at 0.5 mg/mL in that polymeric solution to obtain a second solution (membrane with extract, MBE). AgNO_3_ was simultaneously dissolved in water in different concentrations (1, 3, and 5 mM) and slowly added to that second solution, under mechanical stirring for 24 h at 650 rpm, to prepare three different membrane solutions (MBE1, MBE3, and MBE5), resulting in the generation AgNPs embedded in the polymeric solutions. To obtain the five final membranes (Table 1), each of the prepared solutions, namely, alginate membrane without GPE and AgNO_3_ (MB) as a control, alginate membrane with GPE without AgNO_3_ (MBE) and membranes with GPE and AgNO_3_ at three different concentrations (1, 3, and 5 mM) were then poured into polyethylene Petri dishes (0.21 g/cm^2^). The dishes were dried in a forced‐air Tecnal, ET‐394/3 circulation oven (Piracicaba, São Paulo, Brazil) at 40°C for 24 h.

**TABLE 1:** Composition of the five developed membranes (values in mass for a final volume of 100 mL).

Membranes	Sodium alginate 4% (m/v)	Glycerol 1% (m/v)	GPE 0.5 mg/mL	AgNO_3_		
				1 mM	3 mM	5 mM
MB	4 g	1 g	—	—	—	—
MBE	4 g	1 g	50 mg	—	—	—
MBE1	4 g	1 g	50 mg	17 mg	—	—
MBE2	4 g	1 g	50 mg	—	51 mg	—
MBE3	4 g	1 g	50 mg	—	—	85 mg

### 2.3 Thickness

The thickness of the membranes was determined after dehydration using a digital micrometer (Pantec digital micrometer, resolution 0.001 mm). Three points (*n* = 3) of the membranes were measured in mm, one at the center and the other two around the perimeter [17]. The membrane thickness was expressed as the mean ± standard deviation (SD).

### 2.4 Fourier Transform Infrared (FT‐IR) Spectroscopy

Transmittance FT‐IR was used to assess the presence of chemical groups in the samples. The membranes were previously cut to dimensions of 1 cm × 1 cm and kept in a desiccator with silica for 24 h. Measurements were made on a Fourier transform diffuse reflectance spectrophotometer (Cary 630 Agilent Technologies, Santa Clara, CA, USA) in the wavenumber range of 400 to 4000 cm^−^^1^ with a resolution of 4 cm^−^^1^ during 10 cycles. The FT‐IR spectra were normalized, and the vibration bands associated with the main chemical groups were identified.

### 2.5 Thermal Analyses

Differential scanning calorimetry (DSC) analyses were performed using a DSC‐60 thermal analysis system (Shimadzu, Kyoto, Japan). Samples of approximately 5 mg were weighed into an aluminum pan, sealed, and heated in a DSC furnace at temperatures ranging from 25°C to 350°C, at a heating rate of 5°C/min in an inert atmosphere (N_2_) (45 mL/min). Thermogravimetric analysis (TGA) curves were recorded using a thermoanalytical balance (DTG‐60H, Shimadzu, Kyoto, Japan). Membranes were carefully weighed (5 mg) in a platinum pan and heated to temperatures ranging from 25°C to 800°C (5°C/min) under a dynamic nitrogen atmosphere (50 mL/min).

### 2.6 Mechanical Properties of Membranes

The elongation at break, tensile strength, and Young's modulus were measured using a TA.XT2 texture analyzer (Stable Micro Systems, Surrey, UK) equipped with a 5 Kg load cell. The membranes were cut into 3 cm^2^ sections (1 × 3 cm). The specimens were tested to failure at a crossbar displacement speed of 2 mm/s and a displacement of 40 mm. The analyses were performed in triplicate (*n* = 3).

### 2.7 Water Vapor Permeability

For the water vapor permeability (WVP) measurements, the membranes were sealed onto aluminum cups that contained 20 g of silica gel with an air gap of approximately 6 mm between the desiccant and the film. The cups were then placed into a desiccator cabinet, which contained a solution of saturated NaCl to provide an atmosphere of 75% relative humidity. Air in the cabinet was circulated using a fan, with the temperature of the cabinet maintained at 23°C. Before each weighing, the temperature and humidity of the cabinet were recorded, using an Akso CO277 sensor (São Leopoldo, RS, Brazil). The WVP was then calculated using the following equation [18]:

$$\mathrm{WVP}=\frac{W*e}{\Delta P*t*A}$$

where “*W*” is the weight gain of the cup (g) at the time “*t*” (s), where “*W*/*t*” was determined from the slope of cup weight versus time; “*e*” is the film thickness (mm); “*A*” is the exposed area of the film (m^2^), and “Δ*P*” is the vapor pressure difference across the film based on the relative humidity difference (kPa).

### 2.8 Wettability

Wettability was estimated by determining the contact angle using the Theta Flex Optical Tensiometer (Dyne Testing, Oxfordshire, UK) at controlled temperature of 25°C. A small drop of sample (2–3 µL) was placed on the surface of the device using a microsyringe. The droplet shape is governed by the gravity, density and surface tension of the liquid, and was captured by a high‐resolution digital camera in intervals of 30 ms. The measurement of contact angle was done by the system software.

### 2.9 Irritability Test Using Hen's Egg Chorioallantoic Membrane (HET‐CAM)

Fertilized chicken eggs were procured from a local producer (Fazenda Asa Branca, São Cristóvão, Brazil) immediately after laying and incubated in an automatic rotating incubator for 10 days under controlled conditions of temperature (37.8°C ± 1.0°C) and relative humidity (45%–65%). On the tenth day, the embryo viability was checked to discard any abnormal eggs. The shell of the viable eggs was dissected with forceps, and the inner membrane was meticulously separated to reveal the highly vascularized CAM. The irritation risk of the membranes was evaluated using the Hen's Egg Test on the Chorioallantoic Membrane (HET‐CAM), comparing the results to the negative control (0.9% NaCl) and positive control (0.1 mol/L NaOH), these latter reported before by us [16]. The CAMs were checked for bleeding, coagulation and lysis, and severity scores (S‐scores) were attributed as follows: nonirriting (*S* < 6), mildly irritating (6 ≤ *S* < 12), highly irritating (12 ≤ *S* < 15), and severely irritating (S ≥ 15), and determined using the following equation:

300[IS]=5[301−H]+7[301−L]+9[301−C]

where H is the start time in seconds (s) for the onset of bleeding, L is the start time in seconds (s) for lysis, and C is the start time in seconds (s) for coagulation.

### 2.10 Molecular Docking

The molecular docking study of GPE was conducted using the major compounds: drupanin, artepilin C, and 4,5‐dicaffeoylquinic acid, against a macromolecule belonging to the bacterium *Staphylococcus aureus*, PmtCD [19]. The structural coordinates of the receptors were obtained from the Protein Data Bank [20], using the crystal structure under the code PDB:9Q2Q (PmtCD). The autodock4.exe, autogrid4.exe, and AD4.1_bound.exe files were separated into a folder along with the PDB files of the protein and the molecules of the major compounds. Next, the protein was prepared by removing cocrystallized ligands, free water molecules, and cofactors, leaving only the residues associated with the protein. A grid was defined to surround the region of interest of the active site in the macromolecule, with varying dimensions 70 × 126 × 88, 74 × 126 × 102, and 58 × 126 × 92 Å. Molecular docking calculations were performed using AutoDock Vina and AutoDockTools software (version 1.5.7) [21, 22] and the Discovery Studio viewer. The results were classified according to the values of binding free energy (Δ*G*), binding efficiency, and inhibition constant (*K*_i_) obtained from molecular docking calculations.

### 2.11 Statistical Analysis

Statistical analyses were performed using the GraphPad Prisma software (version 8.0.2). The data presented correspond to the mean ± standard deviation of three independent experiments performed in triplicate and evaluated by one‐way analysis of variance (ANOVA) with Dunnett's post‐hoc test (*p* ≤ 0.05).

## 3 Results and Discussion

### 3.1 Preparation of Polymeric Membranes and Incorporation of AgNPs and GPE

**FIGURE 1:**
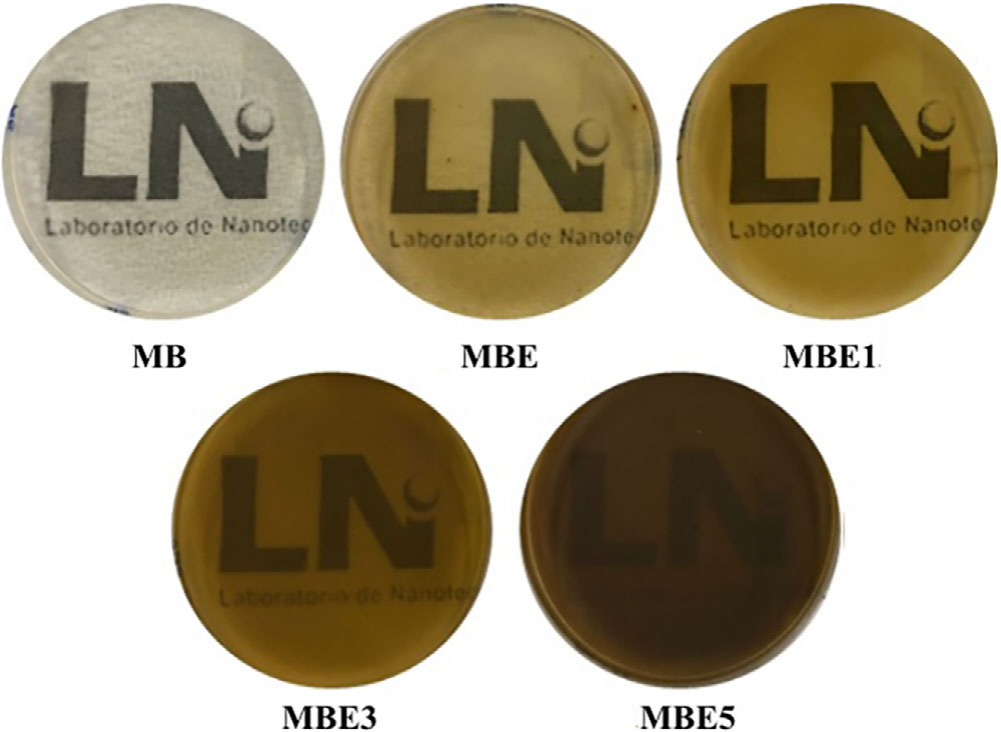
Alginate membrane without GPE and AgNO_3_ (MB), alginate membrane with GPE and without AgNO_3_ (MBE), alginate membrane with GPE and 1 mM AgNO_3_ (MBE1), alginate membrane with GPE, and 3 mM AgNO_3_ (MBE3), alginate membrane with GPE and 5 mM AgNO_3_ (MBE5).

The addition of AgNO_3_ at concentrations of 1.0, 3.0, 5.0 mM, and of GPE, to the solution of sodium alginate and glycerol (MB), led to visible changes in the color from white to dark brown, which suggests the formation of AgNPs (Figure 1). These AgNPs were synthesized by a green method using GPE, in which polyphenols, along with hydroxyl and carboxylic acid groups, facilitated the chelation of Ag^+^. The optical properties were previously analyzed by UV–vis spectroscopy, and the results showed the presence of a plasmonic band associated with the formation of the nanoparticles. The spectra exhibited absorption peaks between 420 and 440 nm, which is indicative of the formation of spheroidal AgNPs [16].

Our findings are consistent with previous studies, demonstrating that propolis‐mediated synthesis reliably produces AgNPs within a comparable size range, regardless of the source or geographical origin of propolis [23]. The nanoparticles synthesized with GPE varying the concentration of AgNO_3_, reported in our previous study [16], revealed a Z‐average size of 115.3 ± 0.71 nm with 1 mM, 109.4 ± 1.12 nm with 3 mM, and 88.48 ± 3.82 nm with 5 mM. The corresponding polydispersity index values (0.339 ± 0.004, 0.229 ± 0.006, and 0.365 ± 0.054, respectively) indicate high uniformity in particle size distribution, aligning with the literature [24, 25, 26]. These consistent results highlight the reliability of propolis as a green synthesis method to obtain AgNPs, reinforcing the potential of GPE as a standardized approach in nanomaterial production.

Five distinct membranes were developed as described in Table 1. All membranes produced with a blend of alginate and glycerol exhibited similar macroscopic characteristics. Images of the membranes produced are shown in Figure 1. The membranes containing different concentrations of AgNO_3_ were investigated and selected based on homogeneity, flexibility, and handling. The membrane containing 3 mM of AgNO_3_ (MBE3) showed the best macroscopic properties, exhibited an excellent balance of strength and flexibility, making it suitable for applications demanding both durability and adaptability as in wounded skin. The remarkable combination of these properties highlights the potential of the membranes, in particular MBE3, for use in fields that require strong yet flexible materials. These findings are particularly relevant for the development of polymeric membranes used in wound dressings, where such characteristics are crucial [27].

### 3.2 Thickness

The membrane thickness analysis revealed a consistent increase of this parameter with the incremental incorporation of the different components. The plain alginate membrane (MB) exhibited the lowest thickness of 0.118 ± 0.001 mm. The addition of GPE to MB resulted in a membrane (MBE) with a slight increase of the thickness to 0.129 ± 0.013 mm. Subsequent incorporation of AgNO_3_ at concentrations of 1 mM (MBE1), 3 mM (MBE3), and 5 mM (MBE5) led to significant increases (*p* ≤ 0.001 for all) in membrane thickness when compared to the control membrane, with MBE5 showing the highest value of 0.174 ± 0.006 mm (Table 2). These results suggest that the addition of GPE and AgNPs contributes to the total membrane thickness, potentially influencing its structural and functional properties.

**TABLE 2:** Assessment of the thickness of the developed membranes. Statistically significant differences between the control membrane (MB) and the MBE, MBE1, MBE3, and MBE5 samples are marked with asterisks: ** *p* ≤ 0.001; **** *p* ≤ 0.0001.

Samples	Thickness (mm)
MB	0.118 ± 0.001
MBE	0.129 ± 0.013
MBE1	0.141 ± 0.004 **
MBE3	0.159 ± 0.011****
MBE5	0.174 ± 0.006****

In the study of Junior et al. [28] membranes obtained with 4% alginate and glycerol, combining with GPE and silica nanoparticles, depicted variations in thickness ranging from 0.131 to 0.143 mm. Notably, the lowest thickness was observed in membranes composed of pure alginate [28]. Our results also align with the study conducted by Dash et al. [29], who reported that increasing AgNP concentration resulted in the increase of membrane thickness, ranging from 214.00 ± 0.97 mm for the formulation containing 0.5% AgNPs to 221.91 ± 0.57 mm for the formulation containing 2.0% AgNPs. This increase was attributed to the enhanced viscosity of the film‐forming solution with the incorporation of nanoparticles.

### 3.3 Fourier Transform Infrared (FT‐IR) Spectroscopy

**FIGURE 2:**
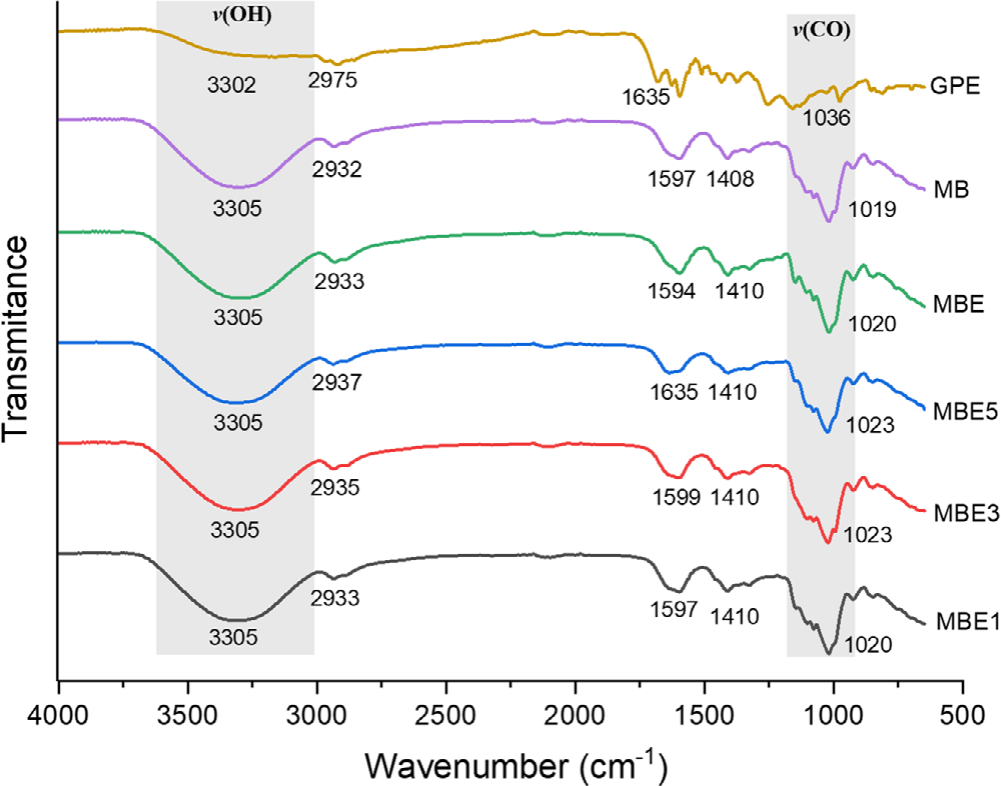
FT‐IR spectra of GPE, plain alginate membrane (MB), alginate membrane with GPE and without AgNO_3_ (MBE), alginate membrane with GPE and 1 mM AgNO_3_ (MBE1), alginate membrane with GPE and 3 mM AgNO_3_, and alginate membrane with GPE and 5 mM AgNO_3_.

FT‐IR spectroscopy of the membranes and extracts was performed to analyze the composition of these materials, evaluate possible interactions, and identify changes in molecular structures. Figure 2. shows the spectra of GPE and the membranes. In the 3600–3300 cm^−^^1^ region of FT‐IR spectra, bands associated with the hydroxyl ν(OH) stretching vibrations were observed at approximately 3302 cm^−^^1^ for GPE, 3305 cm^−^^1^ for MB and MBE, MBE1, MBE3, and MBE5 [5, 30, 31]. The spectra of the membranes showed more defined bands compared to GPE, possibly associated with the hydroxyl groups of glycerol and the presence of residual water. In the 3000–2900 cm^−^^1^ region, vibrational modes related to C─H bond stretching ν(CH) were observed at approximately 2975 cm^−^^1^ for GPE, 2933 cm^−1^ for MB, 2932 cm^−1^ for MBE, 2937 cm^−1^ for MBE1, 2935 cm^−1^ for MBE3, and 2933 cm^−1^ for MBE5 [32].

In the region of 1800–1500 cm^−^^1^, bands related to CC and C═O stretching ν(CC) and ν(CO) were observed, along with COO─type vibrations characteristic of alginate and GPE molecules [28, 30]. Other modes were observed at approximately 1410 cm^−^^1^ associated with C─H bending δ(CH) [33]. The FT‐IR spectra of the samples in the 1300–1010 cm^−^^1^ region showed modes and bands associated with C─O stretching ν(CO), related to the extract and alginate molecules [34, 35], as well as C─O─C ν [36] vibrations specific to the alginate molecule [37, 38, 39]. When evaluating the MBE, MBE1, MBE3, and MBE5 membranes, the spectra showed well‐defined bands, suggesting the incorporation and interaction of the extract with polymers.

### 3.4 Thermal Analyses

**FIGURE 3:**
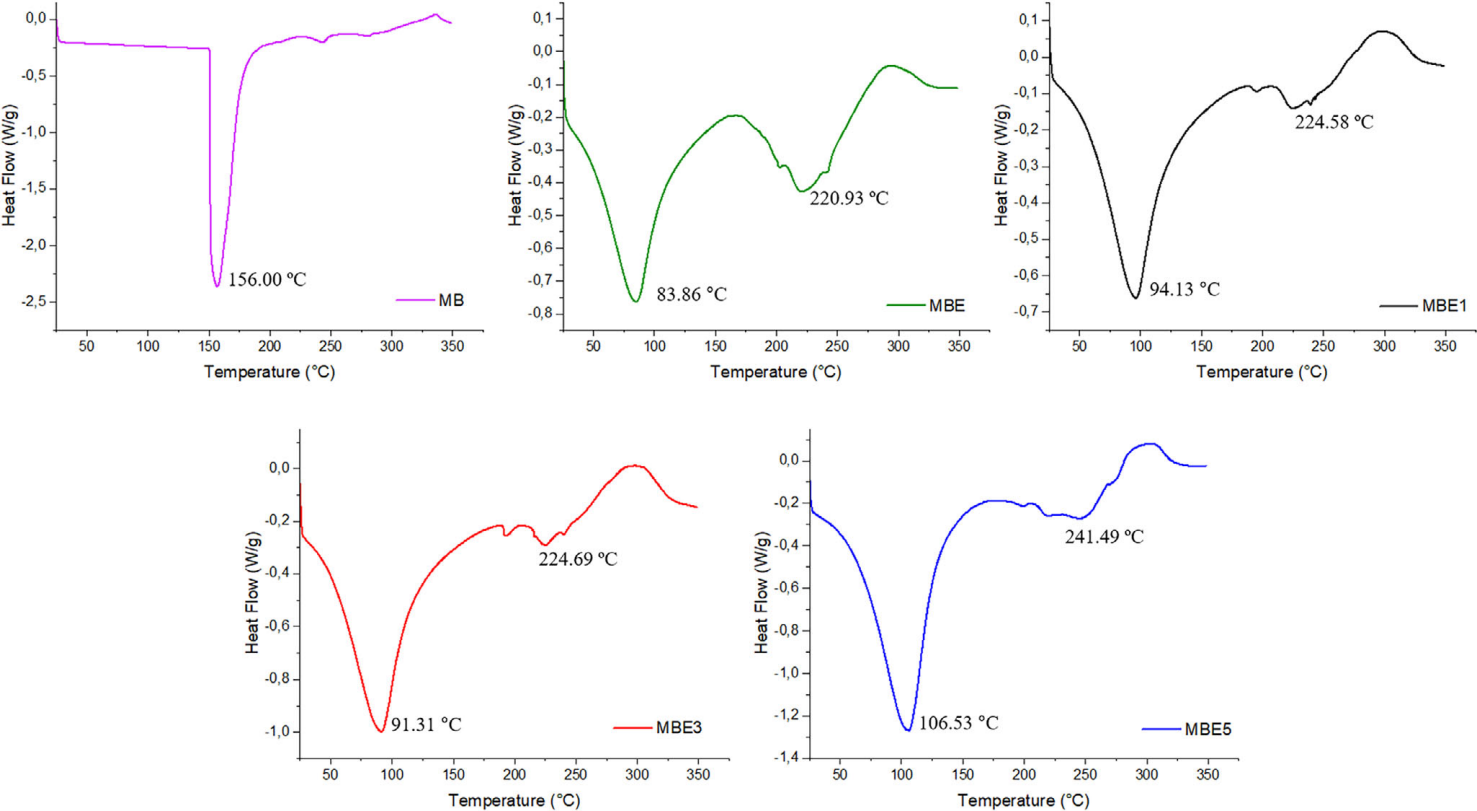
DSC curves of alginate membrane (MB), alginate membrane with GPE and without AgNO_3_ (MBE), alginate membrane with GPE and 1 mM AgNO_3_ (MBE1), alginate membrane with GPE and 3 mM AgNO_3_ (MBE3), and alginate membrane with GPE and 5 mM AgNO_3_ (MBE5). The analysis was carried out over a temperature range of 25°C to 350°C.

A DSC analysis was performed to investigate the endothermic events associated with water loss and thermal transitions of the membranes. Figure 3 shows the DSC curves obtained for MB, MBE, MBE1, MBE3, and MBE5, recorded in a temperature range of 25°C to 350°C.

DSC analysis showed that the MB formulation exhibited an endothermic peak at approximately 156°C, associated with the removal of hydroxyl groups and strongly bound water [40, 41]. In contrast, the DSC curves for the MBE, MBE1, MBE3, and MBE5 formulations showed two well‐defined endothermic events; the first endothermic peak was observed at 83.86°C for MBE, 94.13°C for MBE1, 91.31°C for MBE3, and 106.53°C for MBE5, which is associated with the loss of water and moisture from the membranes [42]. Furthermore, these membranes contain GPE in their composition, which retains physically bound moisture within its internal structure, besides the presence of some easily degradable small molecules, such as organic acids [43].

The second endothermic peak was observed at approximately 220.93°C for MBE, 224.58°C for MBE1, 224.69°C for MBE3, and 241.49°C for MBE5; this event is attributed to the decomposition of the membranes [44]. When comparing the thermogram of MBE with MBE1, MBE3, and MBE5, a modification in the thermograms is observed as the concentration of AgNPs increased. This thermal behavior is in agreement with the studies of Kulkarni et al. [45], showing an increase in the temperature of the endothermic peak with increasing AgNP concentration. Therefore, the increase in the temperature of the endothermic peak may be related to a possible interaction between alginate and GPE‐coated AgNPs.

**FIGURE 4:**
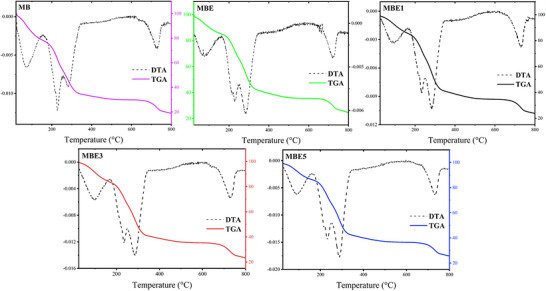
TGA and DTG curves of alginate membrane (MB), alginate membrane with GPE and without AgNO_3_ (MBE), alginate membrane with GPE and 1 mM AgNO_3_ (MBE1), alginate membrane with GPE and 3 mM AgNO_3_ (MBE31), and alginate membrane with GPE and 5 mM AgNO_3_ (MBE5). The analysis was carried out in the temperature range of 25°C to 800°C.

TGA was employed to investigate the effect of AgNPs on the thermal stability of the formulated membranes. In this method, the sample is gradually heated while its mass is monitored, with mass loss indicating physical and chemical changes, such as decomposition, volatilization, and oxidation [28, 46]. By examining the thermogravimetric curve (TGA) and its derivative (DTG), the rate of mass loss over different temperature ranges can be determined, offering detailed insights into the material's thermal properties. This analysis was conducted within a temperature range of 25°C to 800°C, to assess their thermal decomposition behavior and overall stability (Table 3 and Figure 4).

**TABLE 3:** The temperatures at which the MB, MBE, MBE 1, MBE 3, and MBE 5 membranes lost 10%, 20%, 30%, 40%, and 50% of their initial mass, and the percentage of residual mass observed at 800°C.

Samples	T10	T20	T30	T40	T50	Residual mass
MB	73.49°C	133.85°C	208.06°C	234.93°C	268.16°C	18.85%
MBE	100.39°C	203.06°C	235.55°C	269.50°C	295.01°C	24.42%
MBE1	115.40°C	207.43°C	240.02°C	271.35°C	295.60°C	23.66%
MBE3	108.20°C	201.70°C	237.25°C	269.25°C	295.37°C	23.32%
MBE5	111.31°C	209.34°C	241.00°C	274.33°C	295.94°C	25.53%

The first event shown in the spectrum of MB membrane was observed at approximately 72°C, with a mass loss of 6.7%. This event is associated with the removal of free water and water bound to the polymer, as well as the loss of glycerol [47, 48]. This behavior can also be seen in the heat flow analysis, with an endothermic peak at 156°C. The results of the MBE, MBE1, MBE3, and MBE5 membranes for this first event were 75°C, 93°C, 99°C, and 90°C, respectively. For this event, mass losses of 6.8% in MBE, 7.3% in MBE1, 8.5% in MBE3, and 6.9% in MBE5 were observed. This event is related to the removal of water and moisture from the membranes [49].

A second event was displayed by all membranes, observed at temperatures of 228°C, 229°C, 234°C, 234°C, and 232°C, with mass losses of 37.0% for MB, 27.7% for MBE, 27.8% for MBE1, 28.8% for MBE3, and 26.9% for MBE5. This event is attributed to the decomposition of the polymer chain and the breaking of bonds [5, 41]. The results showed a small increase in the temperatures of the membranes with AgNPs, suggesting the interaction of the particles with the polymer. Similar results were observed by Kanagaraj et al. [50], who produced alginate membranes with AgNPs using *Celosia cristata* plant leaf extract and obtained a 2% improvement in membranes’ stability.

The third event occurred at around 721°C for MB, 721°C for MBE, 730°C for MBE1, 728°C for MBE3, and 733°C for MBE5. In this event, mass losses of 76.5% were observed in MB, 70.3% in MBE, 71.7% in MBE1, 71.5% in MBE3, and 70.0% in MBE5. This final event is likely linked to the complete decomposition of the materials and the breaking of the double bonds present in the materials used [51].

### 3.5 Mechanical Properties

**FIGURE 5:**
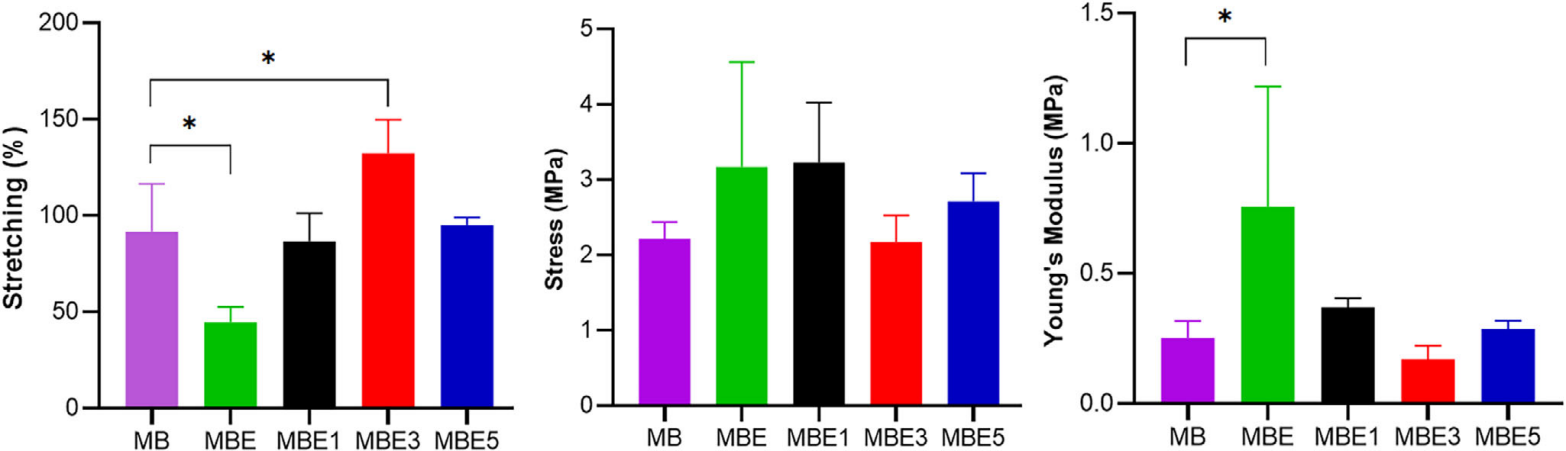
Results of tensile strength, Young's modulus and stress tests for the MB, MBE, MBE1, MBE3, and MBE5 membranes. Statistically significant differences between the control membrane (MB) and the MBE, MBE1, MBE3, and MBE5 samples are marked with asterisks: * *p* ≤ 0.05.

The mechanical properties of the developed membranes, including elongation percentage (which measures the membrane's stretchability before breaking), rupture voltage (which assesses resistance to fracture), and Young's modulus (a measure of stiffness), were evaluated. These key mechanical properties provide insights into the material's flexibility, durability, and structural integrity. The results of these tests are summarized in Figure 5 and Table 4, comparing the performance of the control membrane (MB) with that of the membranes containing extract and nanoparticles (MBE, MBE1, MBE3, and MBE5).

**TABLE 4:** Mechanical properties of the MB, MBE, MBE1, MBE3 and MBE5 membranes. Statistically significant differences between the control membrane (MB) and the MBE, MBE1, MBE3 and MBE5 samples are marked with asterisks: * *p* ≤ 0.05.

	Stretching (%)	Young's modulus (MPa)	Stress (MPa)
MB	85.00 ± 4.36	0.26 ± 0.03	2.18 ± 0.21
MBE	56.00 ± 13.12*	0.48 ± 0.03*	2.70 ± 0.80
MBE1	90.67 ± 9.02	0.32 ± 0.01	2.94 ± 0.31
MBE3	96.67 ± 11.55*	0.29 ± 0.03	2.78 ± 0.13
MBE5	95.00 ± 4.00	0.29 ± 0.04	2.71 ± 0.37

The stretching assessment showed a value of 85% for MB. This high value may be associated with the presence of glycerol in the formulation, which resulted in reduced stiffness due to a decrease in interactions within the alginate chain, owing to its role as a plasticizer [52]. This occurs because the presence of a plasticizer, such as the glycerol used in this study, forms strong hydrogen bonds, which increases intramolecular spacing, resulting in a reduction in internal hydrogen bonds [53].

When comparing MB with MBE, a reduction in flexibility was observed for the MBE membrane. This behavior may be attributed to interactions between bioactive compounds present in GPE and the alginate polymeric chains, which limit chain mobility within the membrane matrix. As a result, stronger intermolecular interactions may have contributed to the reduced flexibility observed for MBE [44, 54].

When nanoparticles were incorporated into the membranes at different concentrations (MBE1, MBE3, and MBE5), an increase in the stretching was observed, with MBE3 showing the most significant increase at 96.67%. The Young's modulus results showed higher values for the MBE, MBE1, MBE2, and MBE5 membranes when compared with MB. A possible interaction between the extract and the nanoparticle with the membranes is suggested, resulting in less rigid membranes. The stress values were below 3 MPa for all membranes, with the highest value for MBE1 (2.78 MPa).

It is therefore suggested that incorporating AgNPs resulted in membranes that were more flexible and more resistant to deformation. The study by Gudimalla et al. [55] observed that the incorporation of AgNPs into their films increased the elongation at break without compromising tensile strength, resulting in films with good strength and flexibility.

Therefore, the results obtained in these studies suggest that the addition of AgNPs and the extract appears to enhance the mechanical properties of the membranes, representing a viable strategy for creating more flexible alginate dressings capable of adapting to skin movements; these properties are important for a potential application in wound healing.

### 3.6 Water Vapor Permeability

**FIGURE 6:**
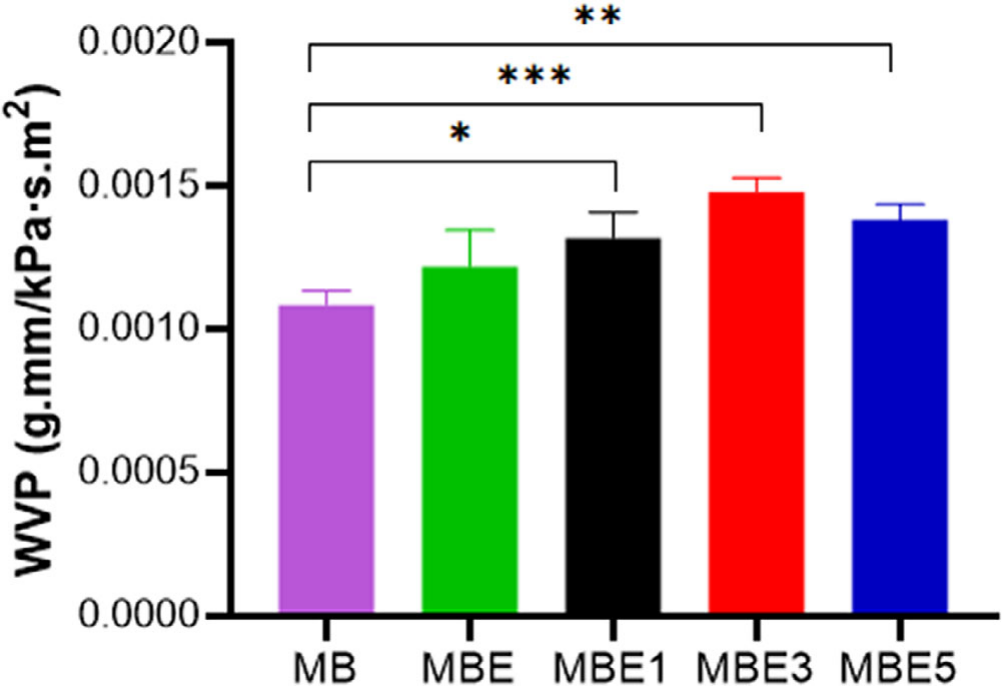
Water vapor permeability (WVP) results for alginate membranes (MB), alginate membranes containing GPE and without AgNO_3_(MBE), and alginate membranes containing AgNPs produced using concentrations of 1 mM (MBE1), 3 mM (MBE3), and 5 mM (MBE5) AgNO_3_. Statistically significant differences between the control membrane (MB) and the MBE, MBE1, MBE3, and MBE5 samples are marked with asterisks: * *p* ≤ 0.05; ** *p* ≤ 0.01; *** *p* ≤ 0.001.

WVP is an important property for the analysis of polymer‐based wound dressings as it translates the ability of the membranes to regulate moisture and avoid the accumulation of excess exudate in wounds [56]. The present work compared the WVP values of alginate membranes with and without the incorporated GPE and AgNPs (Figure 6 and Table 5).

**TABLE 5:** Water vapor permeability (WVP) results for the MB, MBE, MBE1, MBE3, and MBE5 membranes. Statistically significant differences between the control membrane (MB) and the MBE, MBE1, MBE3, and MBE5 samples are marked with asterisks: * *p* ≤ 0,05; ** *p* ≤ 0.01; *** *p* ≤ 0.001.

Samples	WVP (10^−3^g.mm/kPa∙s∙m^2^)
MB	1.08 ± 0.05
MBE	1.22 ± 0.13
MBE1	1.32 ± 0.09^*^
MBE3	1.47 ± 0.05^***^
MBE5	1.38 ± 0.05^**^

The WVP value for the MB membrane was 1.08 × 10^−^^3^g.mm/kPa∙s∙m^2^ this value may be attributed to the presence of glycerol in the composition, which caused spacing between the chains [57]. The MBE result showed an increase in the value (1.22 × 10^−^^3^ g.mm/kPa∙s∙m^2^) compared with MB, suggesting the incorporation of the extract into the membrane. When comparing MB with MBE1, MBE3, and MBE5, significant increases were observed, with *p* ≤ 0.05 for MBE1, *p* ≤ 0.001 for MBE3, and *p* ≤ 0.01 for MBE5.

This increase is attributed to the incorporation of AgNPs into the alginate matrix, reducing interactions between the polymeric chains, promoting the formation of voids and increasing the permeability of the membrane. High WVP values may contribute to the wound healing process, whilst lower values may lead to increased wound exudation [58]. Thus, membranes containing nanoparticles exhibit improved properties for potential use as wound dressings.

Similar results regarding the increase in WVP were obtained by Martínez–Molina et al. [59] for alginate membranes with AgNPs synthesized by green methods. The WVP values for their alginate‐based edible membranes from *Sargassum fluitans* ranged from 0.83 to 1.74 g.mm/h.kPa.m^2^, indicating that the incorporation of AgNPs influenced the membrane's permeability. Optimizing the composition and concentration of these bioactives is crucial for developing effective wound dressings. The ability to modulate WVP through biosynthesized AgNPs offers opportunities for creating healing biomaterials that maintain optimal moisture while providing antimicrobial protection [60].

### 3.7 Wettability

**FIGURE 7:**
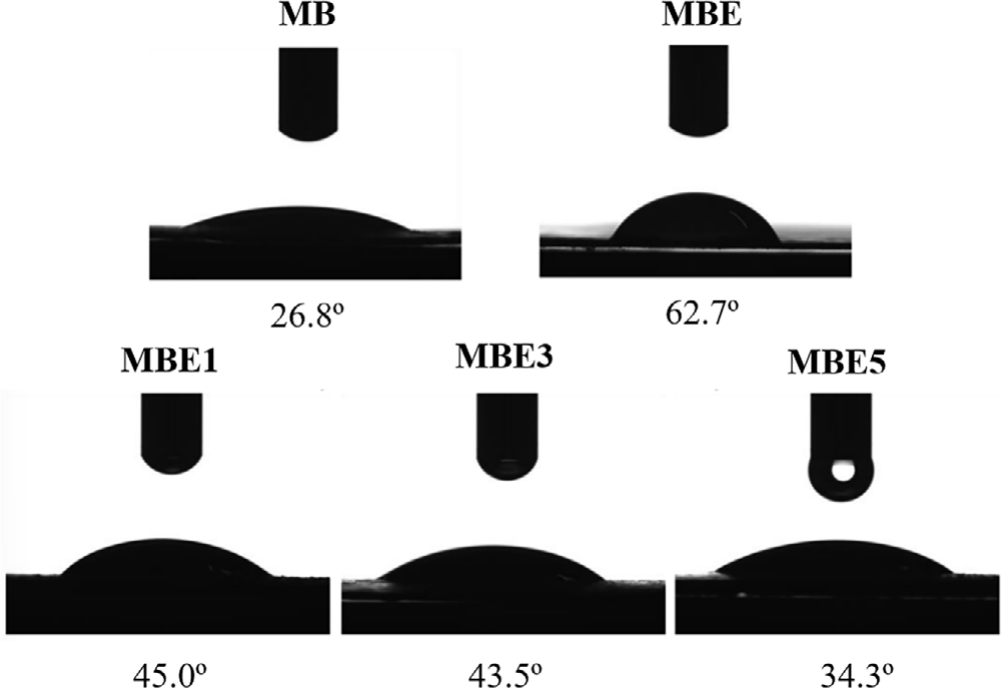
Analysis in wettability of membranes through of angle in contact. Alginate membrane (MB), alginate membrane with GPE and without AgNO_3_(MBE), and alginate membranes containing green AgNPs at concentrations 1 mM (MBE1), 3 mM (MBE3), and 5 mM (MBE5) of AgNO_3_.

The surface wettability of the developed alginate membranes was analyzed using a tensiometer to measure contact angles (Figure 7). Lower contact angles (<90°) indicate increased hydrophilicity, while higher angles (>90°) reflect hydrophobic tendencies. All measurements were performed at a controlled temperature of 25°C, and all membranes exhibited high wettability, with contact angles of 26.8° for the plain membrane (MB), 62.7° for the membrane with GPE (MBE), and 45.0°, 43.5°, and 34.3° for MBE1, MBE3, and MBE5, respectively.

The study by Aburabie, Eskhan and Hashaikeh [61] involved sodium alginate cross‐linked with calcium chloride (CaCl_2_), which resulted in high hydrophilicity with a contact angle of 25°. However, our work achieved similar hydrophilic properties without the need for cross‐linking agents like CaCl_2_. This highlights a key difference, as the membranes kept their hydrophilicity, while eliminating the additional step of chemical cross‐linking, simplifying the formulation process and potentially broadening the applications of the developed materials.

When comparing MB with MBE, a 134% increase in contact angle values was observed. When comparing MB with membranes containing nanoparticles, an increase of 67.91% was observed for MBE1, 62.31% for MBE3, and 27.99% for MBE5. Even with this increase, all membranes presented angles smaller than 90°C, resulting only in hydrophilic membranes. This result can be linked to the presence of organic compounds in the biosynthesized AgNPs with GPE. The combination of organic molecules and polysaccharides influences the surface of the material, potentially enhancing the antimicrobial effectiveness of the membranes by helping repel microorganisms and reduce the risk of infection [62, 63].

### 3.8 Irritability Test Using Hen's Egg Chorioallantoic Membrane (HET‐CAM)

**FIGURE 8:**
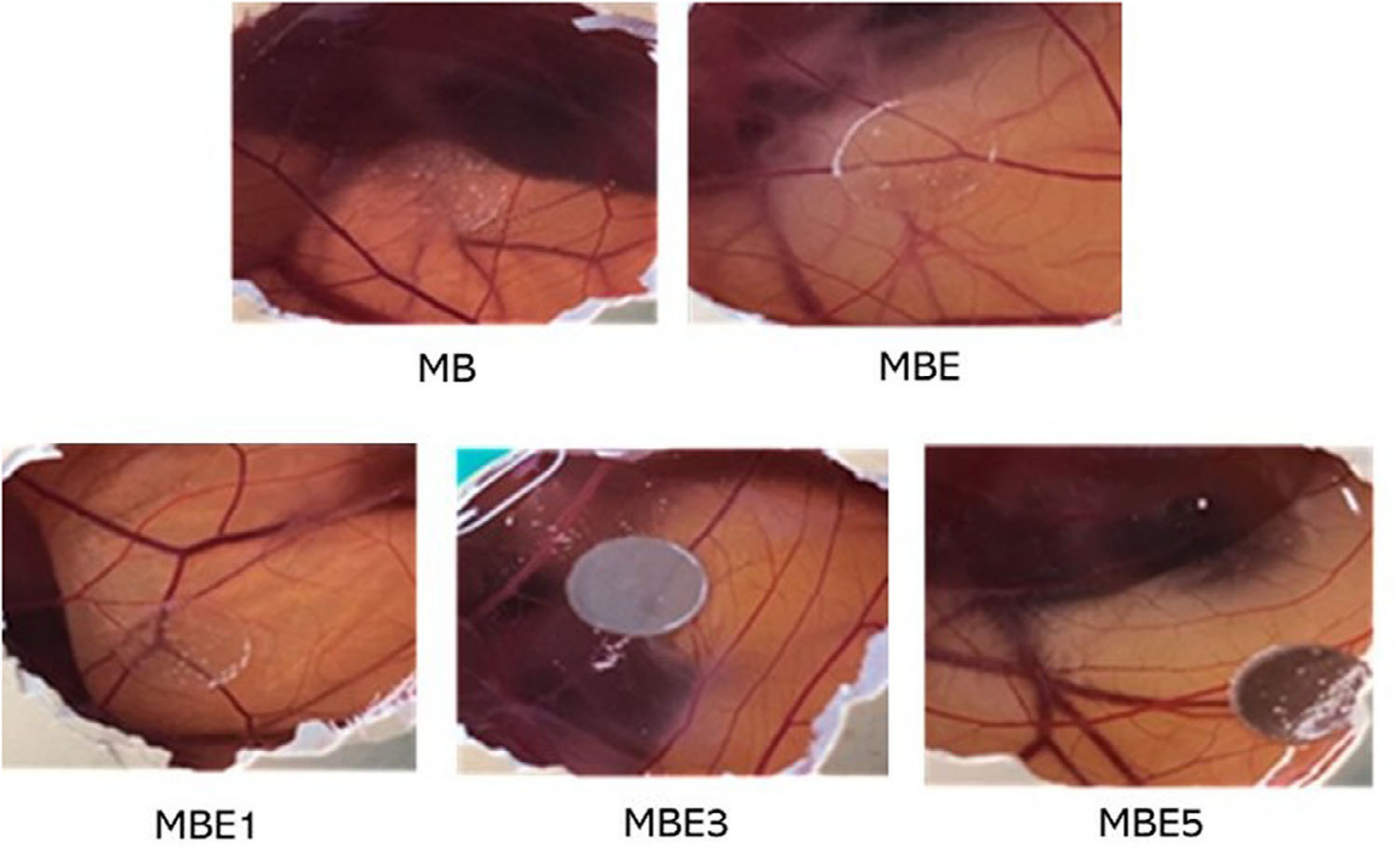
HET‐CAM analysis of the membranes developed, namely the plain alginate membrane (MB), alginate membranes with GPE and without AgNO_3_(MBE) and alginate membranes containing biosynthesized AgNPs with GPE, and different concentrations of AgNO_3_ (MBE1, MBE3, and MBE5).

The HET‐CAM assay is a recognized method for assessing the irritancy of drug delivery systems, including AgNPs synthesized from GPE, on the CAM [64]. The results of this analysis are seen in Table 6 and Figure 8.

**TABLE 6:** HET‐CAM analysis scores for the MB, MBE, MBE1, MBE3 and MBE5 membranes, and for the negative (0.9% NaCl) and positive (0.1 M NaOH) controls. The score indicates: *S* < 6 for nonirritating, 6 ≤ *S* < 12 for slightly irritating, 12 ≤ *S* < 15 for highly irritating, and S ≥ 15 for severely irritating.

Samples	Irritation index
Positive control	16
Negative control	0
MB	0
MBE1	0
MBE3	0
MBE5	0

The results showed that all the membranes tested were nonirritating, highlighting the potential of these membranes for use as dressings without causing adverse reactions [65]. This study provided a preliminary assessment of the membranes’ biocompatibility, cytotoxicity assays are still required for further validation.

Vellingiri et al. [66] described the anti‐angiogenic efficacy of silver oxide nanoparticles (Ag_2_ONPs) obtained from endophytic fungus, and the use of HET‐CAM analysis to confirm the nonirritating properties of the Ag_2_ONPs, making them promise for biomedical applications, especially in metastasis prevention. Similarly, the safety of alginate has been validated in ophthalmic gel formulations [67] and in nanogels for sustained drug release [68] using the HET‐CAM. These results highlight the potential of both AgNPs and alginate as nonirritating and nontoxic materials. However, further analysis, such as cytocompatibility and safety tests, are required to verify the biocompatibility of these materials for the development of drug delivery systems.

### 3.9 Molecular Docking

Skin infections can be caused by bacteria (e.g., *Staphylococcus* spp.) [69] invading normal skin or affecting a compromised skin barrier (e.g., atopic dermatitis or surgical wound sites) [70]; for this reason, PmtCD protein has been selected as target in molecular docking studies.

**FIGURE 9:**
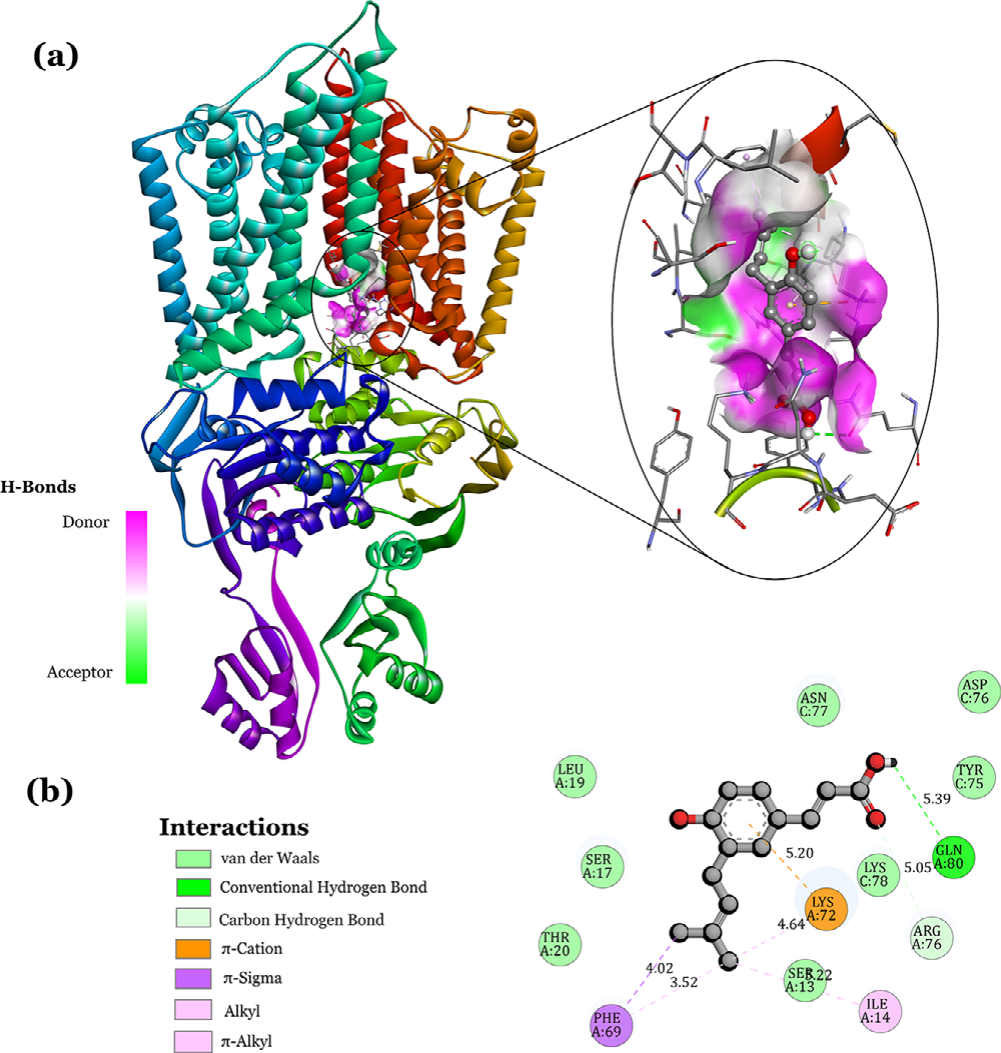
(a) Molecular coupling of the PmtCD protein with drupanin. (b) 2D interaction map showing the specific amino acid residues involved in the interaction of drupanin with PmtCD.

Molecular docking analysis revealed that drupanin exhibits excellent affinity for PmtCD (Figure 9a), with a binding free energy (Δ*G*) of −6.34 kcal/mol, ligand efficiency of −0.37 kcal/mol, and an inhibition constant of 22.67 µM. In thermodynamic terms, the PmtCD‐drupanin (protein‐ligand) interaction is stable and occurs spontaneously. This stability is due to the interactions that occur between the atoms of drupanin and the residues belonging to the PmtCD protein (Figure 9b). Drupanin is stabilized by a lattice of non‐covalent interactions, van der Waals interactions (LEU:19, SER:17, THR:20, SER:13, LYS:78, TYR:75, ASP:76, and ASN:77), conventional hydrogen bonds (GLN:80), carbon‐hydrogen bond (ARG:76) interacting with the C═O of drupanin, in addition to π‐cation interaction (LYS:72) with the aromatic ring, π‐sigma (PHE:69), alkyl, and π‐alkyl (PHE:69, ILE:14) which reinforce stability at the active site.

**FIGURE 10:**
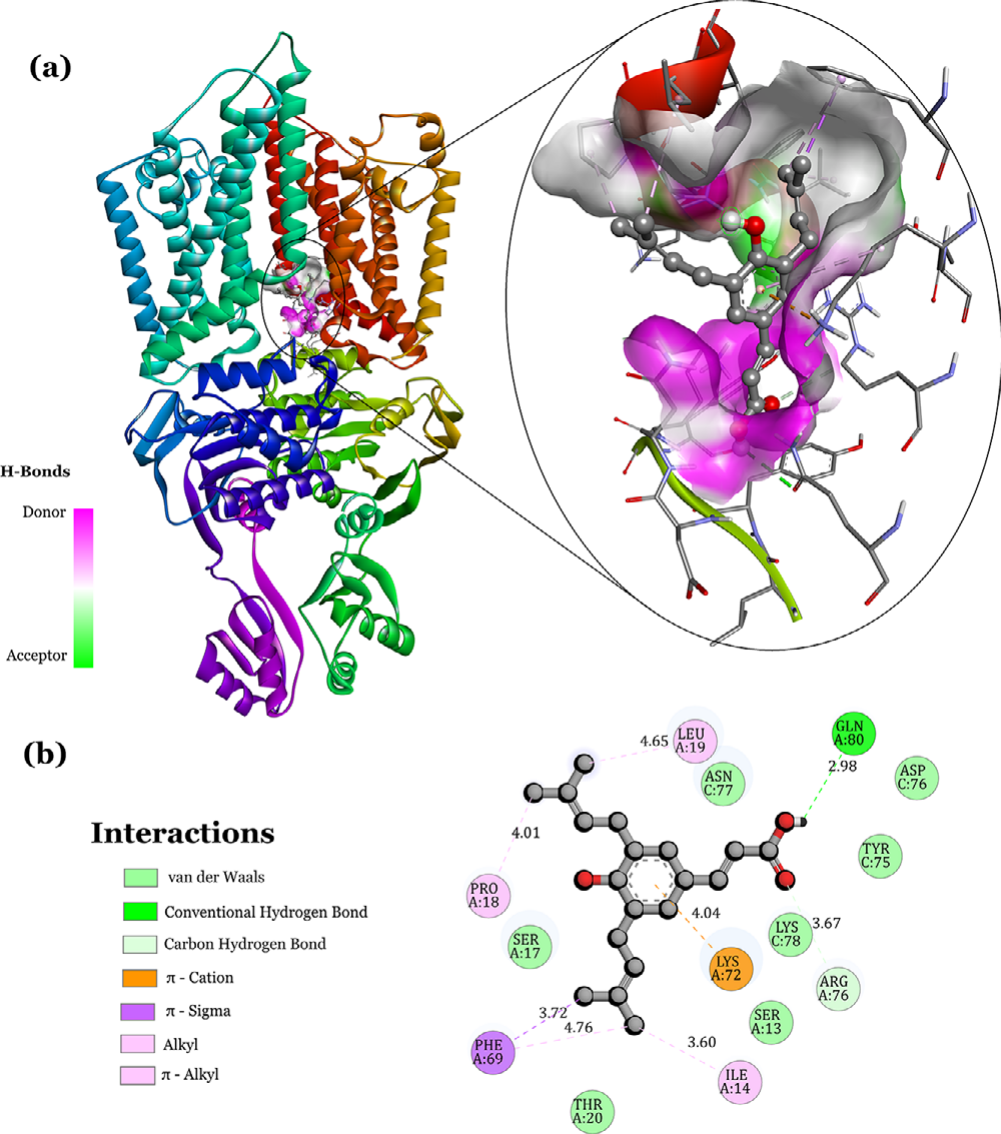
(a) Molecular coupling of the PmtCD protein with artepillin C. (b) 2D interaction map showing the specific amino acid residues involved in the interaction of artepillin C with PmtCD.

Molecular coupling with the artepilin C molecule exhibits a favorable affinity for PmtCD (Figure 10a), with a binding free energy (Δ*G*) of −5.89 kcal/mol, ligand efficiency of −0.27 kcal/mol, and an inhibition constant of 48.46 µM, showing a stable and spontaneous PmtCD‐artepilin C (protein–ligand) interaction. This stability is attributed to the interactions between the atoms of artepillin C and the residues belonging to the PmtCD protein (Figure 10b). Artepillin C is stabilized by a lattice of non‐covalent interactions, similar to the PmtCD‐drupanin interaction, since both are compounds present in GPE. These are the van der Waals interactions (SER:17, THR:20, SER:13, LYS:78, TYR:75, ASP:76, and ASN:77), conventional hydrogen bonds (GLN:80), carbon─hydrogen bond (ARG:76) interacting with C═O of artepilin C, and π‐cation (LYS:72), π‐sigma (PHE:69), alkyl, and π‐alkyl (PRO:18, LEU:19, ILE:14) interactions.

**FIGURE 11:**
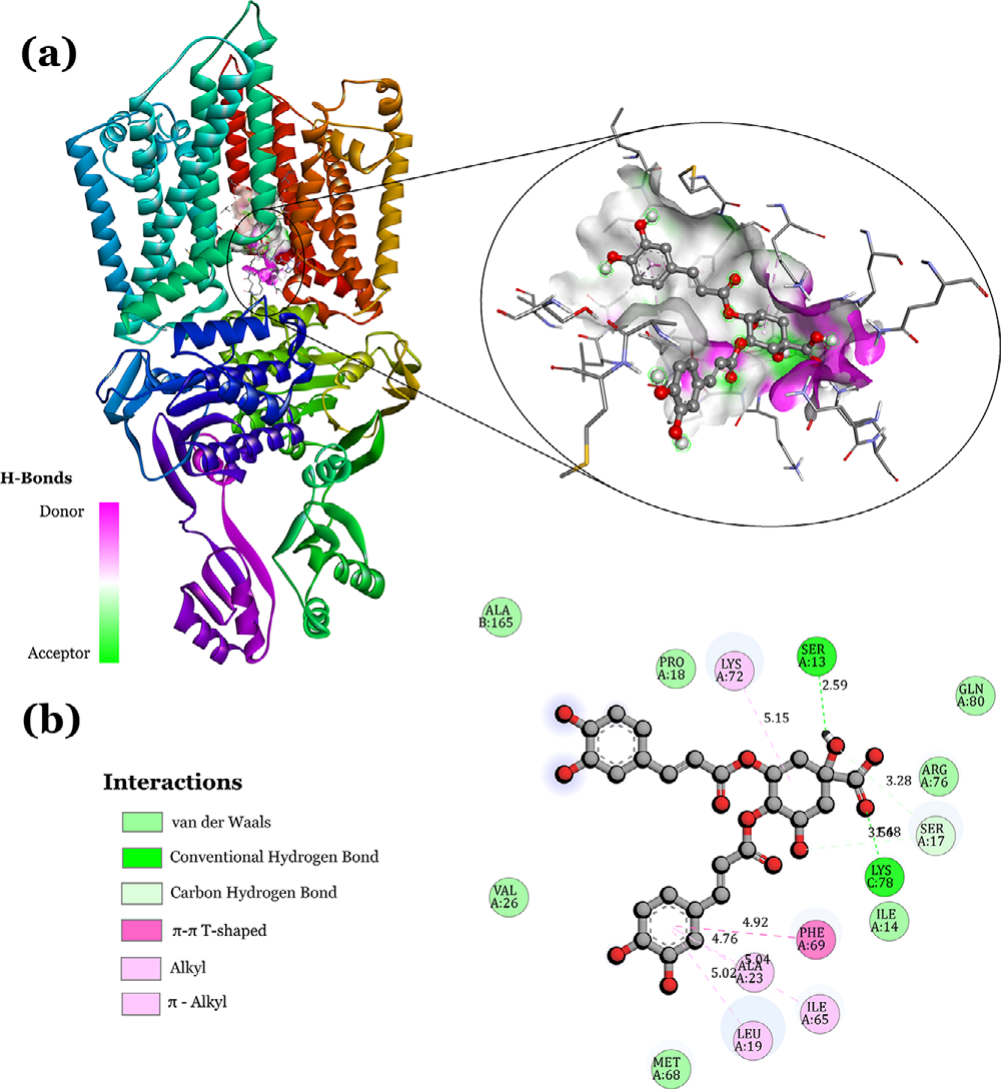
(a) Molecular coupling of the PmtCD protein with 4,5‐dicaffeoylquinic acid. (b) 2D interaction map showing the specific amino acid residues involved in the interaction of 4,5‐dicaffeoylquinic acid with PmtCD.

In parallel, the macromolecule PmtCD showed a reasonable interaction with 4,5‐dicaffeoylquinic acid (Figure 11a). The stability of this coupling is ensured by a lattice of specific interactions to anchor 4,5‐dicaffeoylquinic acid to the active site, observed in the 2D interaction map (Figure 11b). Interactions like van der Waals type (ALA:165, VAL:26, MET:68, ILE:14, ARG:76, GLN:80, and PRO:18), conventional hydrogen bonding (SER:13, LYS:78), π–π T‐shaped (PHE:69), Alkyl and π‐Alkyl (LYS:72, ALA:23, LEU:19, and ILE:65) are seen. The molecular interaction resulting from 4,5‐dicaffeoylquinic acid with PmtCD showed reasonable thermodynamic values, although slightly lower than that observed for the other two molecules. The coupling exhibited a binding free energy (Δ*G*) of −4.8 kcal/mol, indicating spontaneous binding.

Thus, molecular docking data suggest that the major compounds of green propolis have the potential to inhibit the proliferation of *Staphylococcus aureus* bacteria (known to cause nosocomial infections) [69, 70]. The favorable affinity shown by drupanin (Δ*G* = −6.34 kcal/mol) indicates that propolis‐coated nanoparticles can interact directly with the bacterial membrane and inhibit its functions, since this protein helps the bacteria to “attack” the human immune system.

## 4 Conclusions

The incorporation of AgNPs into alginate membranes resulted in notable changes in the physicochemical and structural properties of the final membranes, making them suitable for use as wound dressings. The membranes exhibited a color change to brownish after the addition of GPE and AgNPs to plain MB, indicating interaction between the components. In addition, an increase in thickness was observed, with the highest value for MBE3 (0.159 mm), as well as improved mechanical properties with increased Young's modulus, tensile strength, and stress, resulting in more flexible membranes when AgNPs were present. Furthermore, an improvement in barrier performance was observed in the analysis of WVP, contributing to the potential use of MBE3 as a wound dressing.

Other properties, such as thermal stability, were improved, indicating that AgNPs positively impact the overall durability and functionality of the membranes. HET‐CAM analysis confirmed that the membranes do not cause irritation, which is a promising result. Furthermore, through molecular docking, it was observed that molecules, such as artepilin C, 4,5‐dicaffeoylquinic acid, and drupanin present in GPE can interact directly with the bacterial membrane and inhibit its functions, since this protein helps bacteria “attack” the human immune system. The interactions of the molecules derived from GPE compounds were spontaneous with the macromolecular residues, showing potential to inhibit the proliferation of *Staphylococcus aureus* bacteria. These findings suggest the potential of alginate membranes with AgNPs in applications requiring greater mechanical strength, stability, and nonirritating properties, making them a promising material for use in dressings.

## Author Contributions

**Erica Tirzah S. Lima**, **Victoria L. S. Santos**, **Jad Lorena F. Simplício**, **Erika S. Lisboa**, **Sona Jain**, **Eliana B. Souto**, and **Patrícia Severino** contributed for the conception and design of this study, for the analysis and interpretation of the experimental data, for the drafting of the original paper by revising it critically for intellectual content. All authors approve the final version of the manuscript to be published; and that all authors agree to be accountable for all aspects of the work.

## Ethics Statement

This authors have nothing to report.

## Conflicts of Interest

The authors declare no conflicts of interest.

## Data Availability

The data that support the findings of this study are available from the corresponding author upon reasonable request.
